# Supplementary material for: Association Between Active DNA Demethylation and Liver Fibrosis in Individuals with Metabolic-Associated Steatotic Liver Disease (MASLD)
Source: Int J Mol Sci. 2025 Jan 31;26(3):1271. doi: 10.3390/ijms26031271 (PMC11818491; doi:10.3390/ijms26031271)
Supplement: Supplementary file 1 [file ijms-26-01271-s001.zip › ijms-3417094-supplementary.pdf]

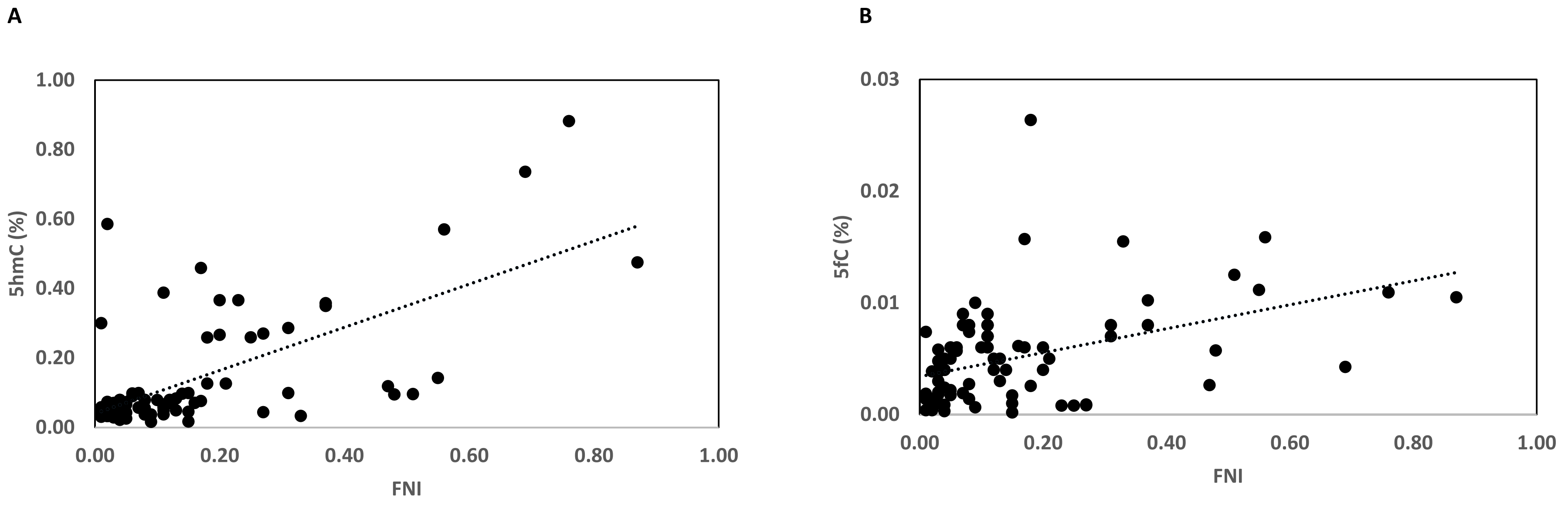

Supplementary Figure S1. Scatter plot for the relationship of FNI with global 5hmC (A) and 5fC (B).

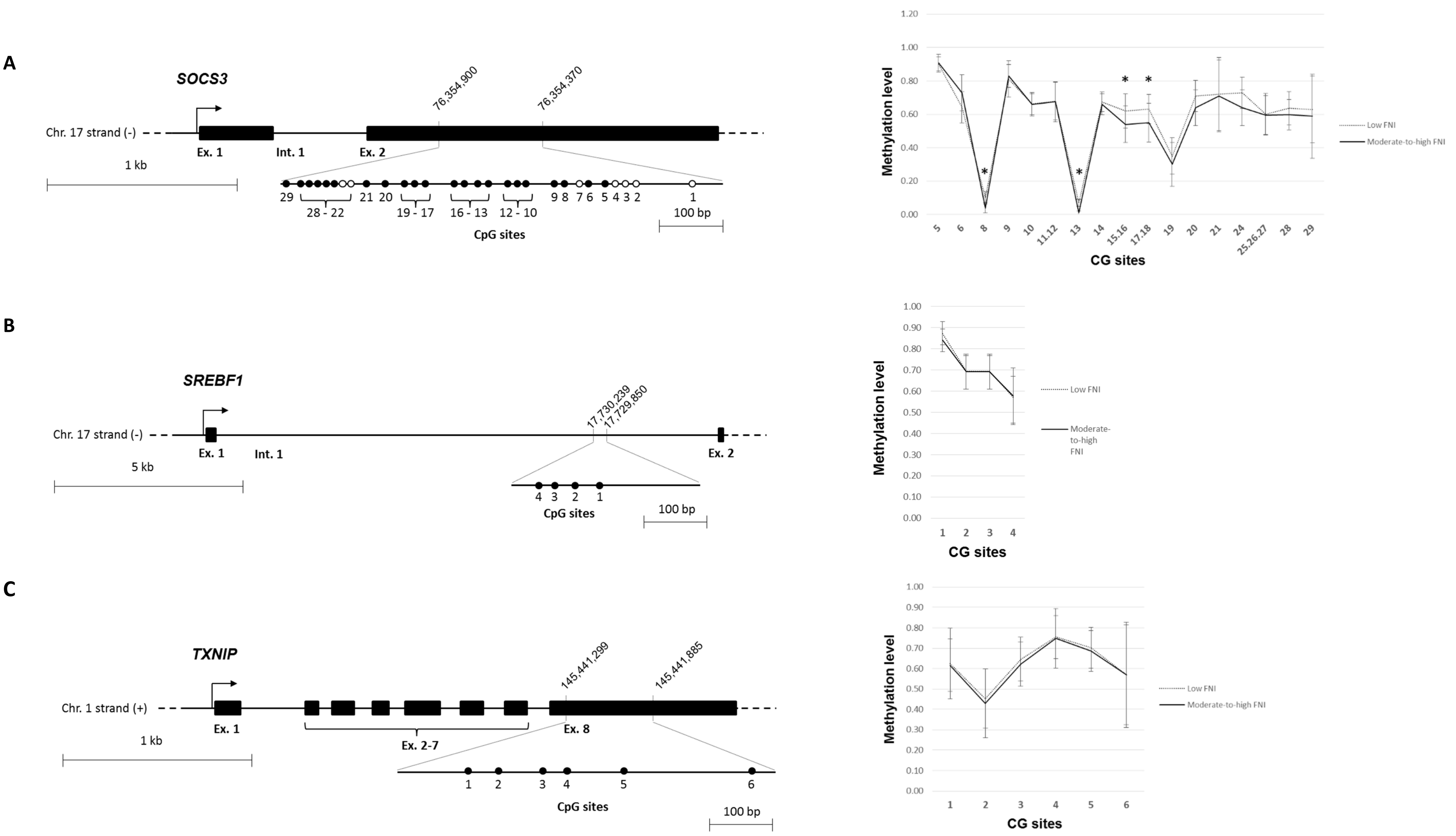

Supplementary Figure S2. DNA methylation profiles of SOCS3 (A), SREBF1 (B) and TXNIP (C) in low and moderate-to-high FNI individuals. Left panels show schematic representation of the CpG sites distribution within the DNA regions under investigation. The images indicate the location and extent of the regions analyzed in the EpiTYPER assay (filled circles represent the analyzed CpG sites). Filled boxes represent exons. The genomic positions refer to the 2009 (GRCh37/hg19) assembly. Right panels show CG methylation level in low and moderate-to-high FNI individuals. Data are mean ± SD. * $p < 0.05$ (Student t-test).

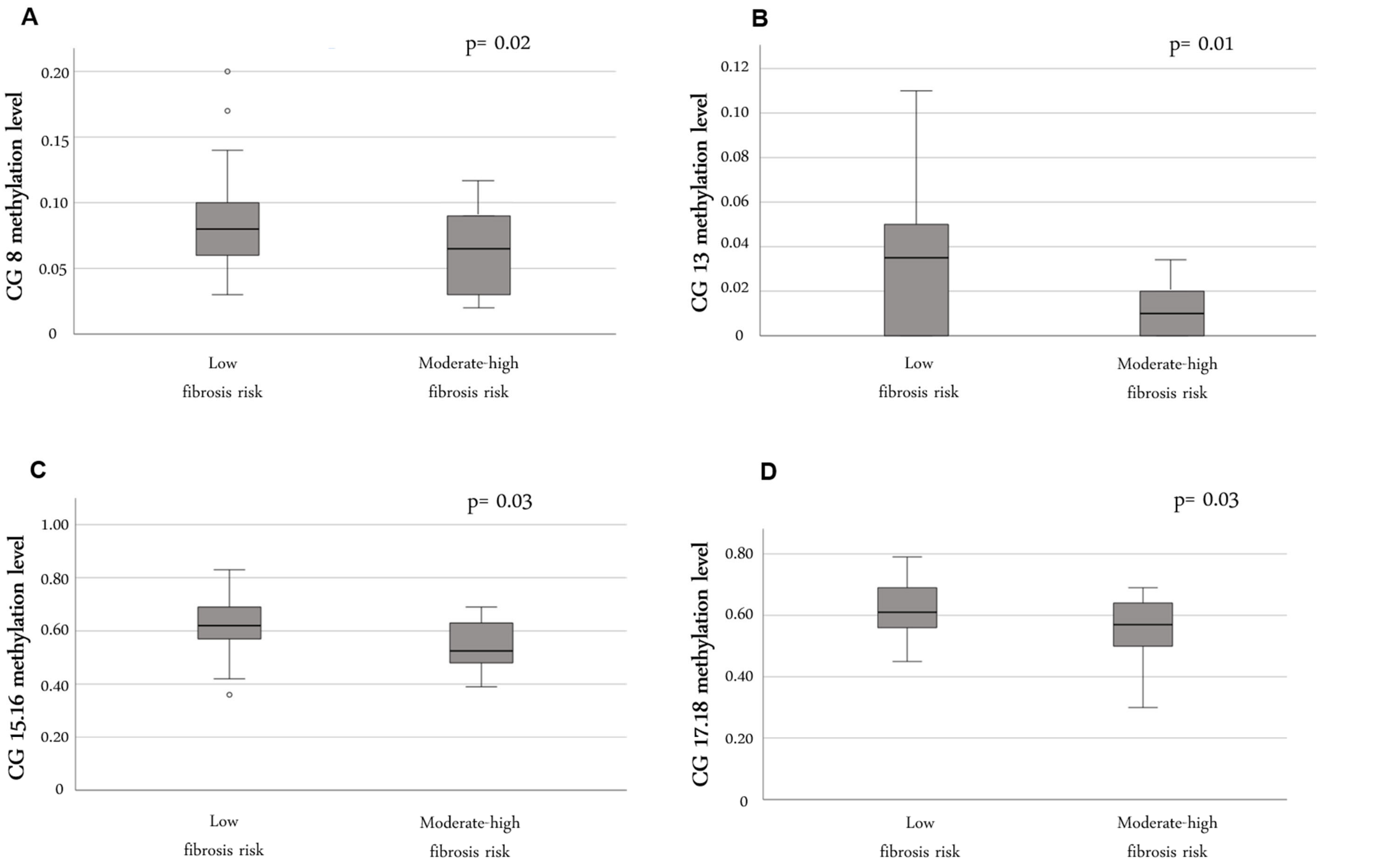

Supplementary Figure S3. DNA methylation profiles of SOCS3 CpG 8 (A), 13 (B), 15.16 (C) and 17.18 (D) in individuals at low and moderate-to-high fibrosis risk. Median (95% C.I.) levels of the DNA methylation level according to the fibrosis risk (low risk: FNI < 0.10; moderate to-high risk: ≥ 0.1). Values more than 1.5 x interquartile range are represented by circles. p-values from Student's T-test are reported.

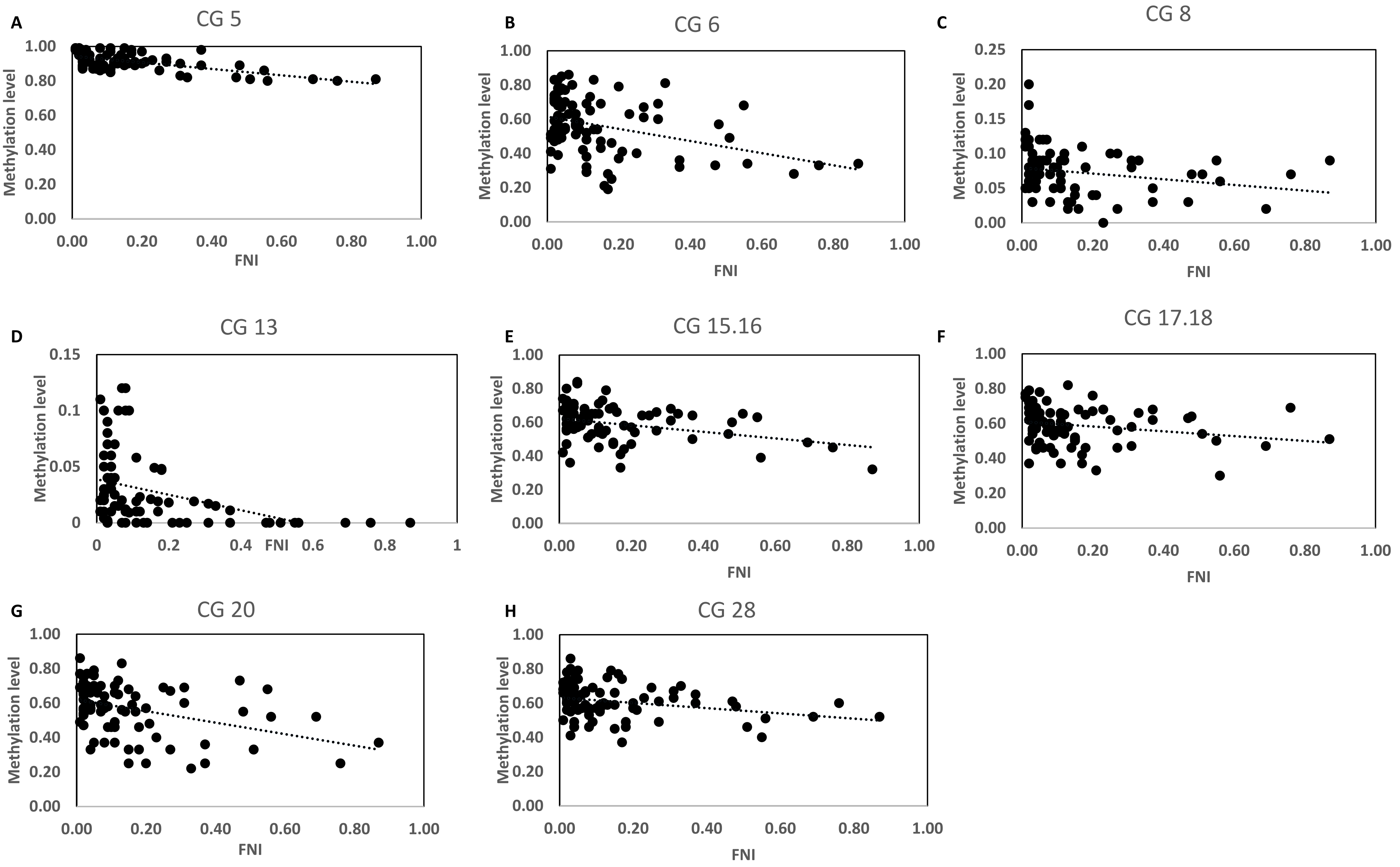

Supplementary Figure S4. Scatter plot for the relationship of FNI with DNA methylation of CpG 5 (A), 6 (B), 8 (C), 13 (D), 15.16 (E), 17.18 (F), 20 (G) and 28 (H) of the SOCS3 locus

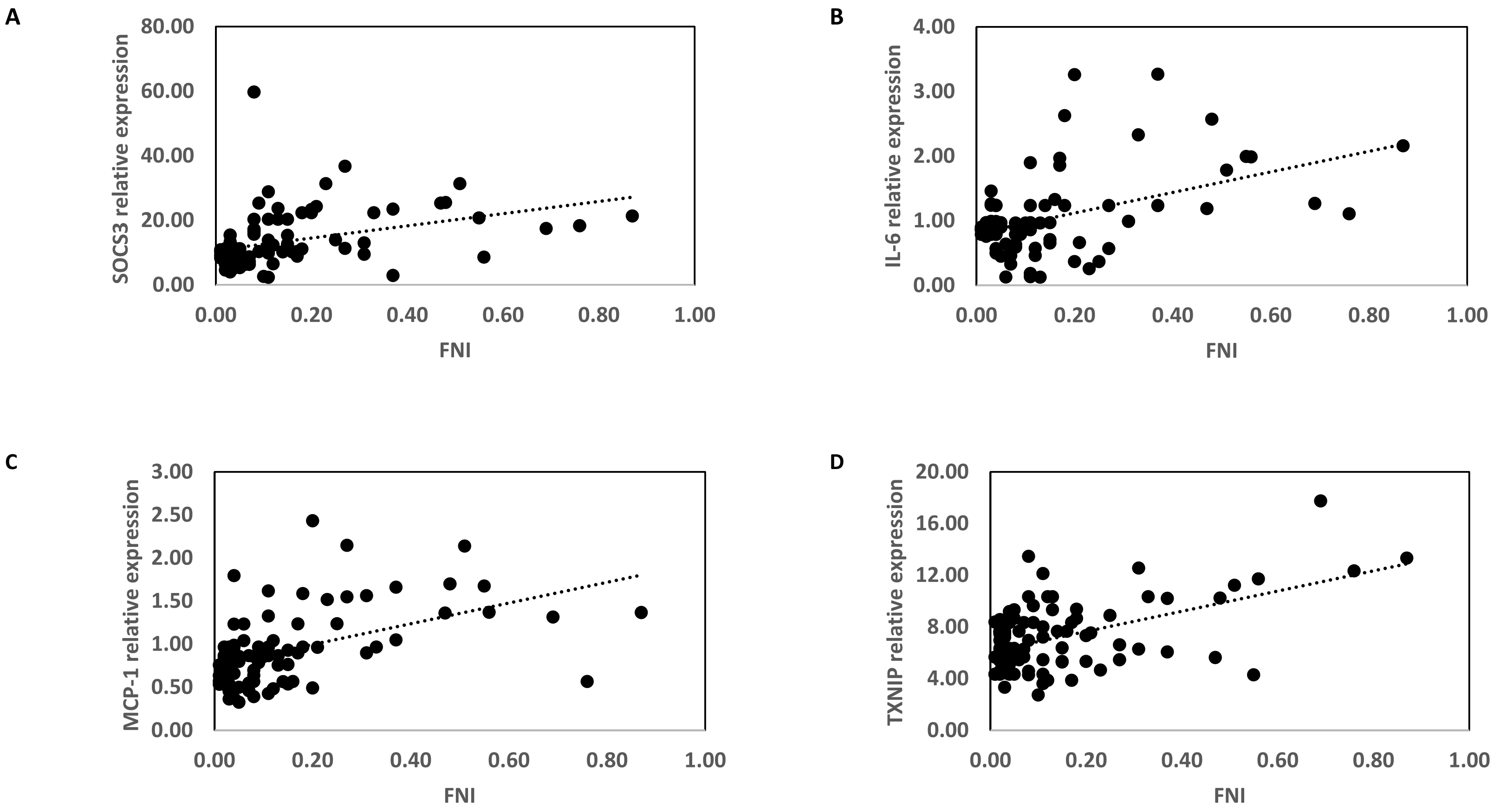

Supplementary Figure S5. Scatter plot for the relationship of FNI with mRNA expression level of SOCS3 (A), IL-6 (B), MCP1 (C) and TXNIP (D).

A

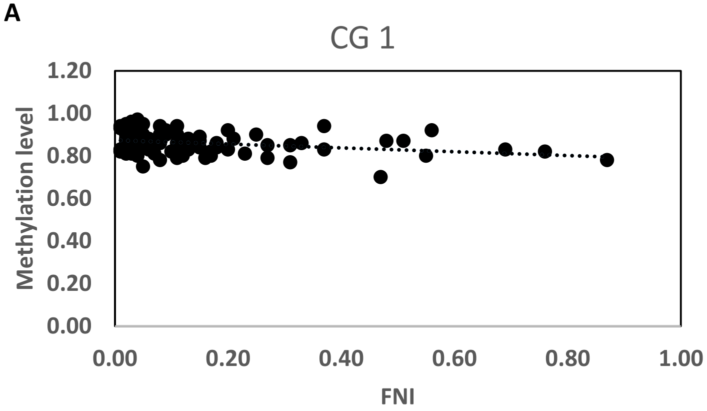

B

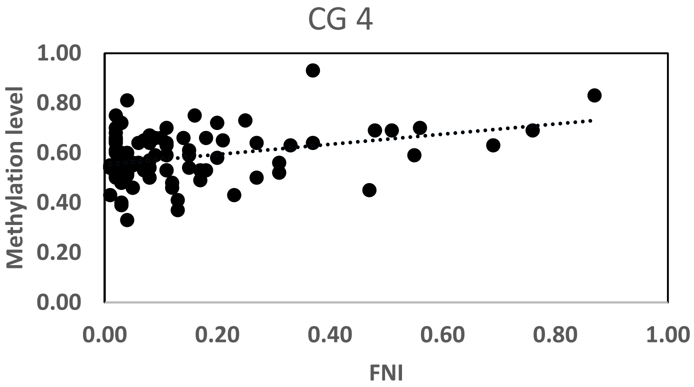

Supplementary Figure S6. Scatter plot for the relationship of FNI with DNA methylation of GpG 1 (A) and 4 (B) of the SREBF1 locus
